# Machine Learning-Assisted Rapid Optical Imaging for Label-Free CAR T-Cell Detection in Whole Blood

**DOI:** 10.3390/bios16050240

**Published:** 2026-04-24

**Authors:** Nanxi Yu, Ryan M. Porter, Xinyu Zhou, Wenwen Jing, Fenni Zhang, Eider F. Moreno Cortes, Paula A. Lengerke Diaz, Jose V. Forero Forero, Erica Forzani, Januario E. Castro, Shaopeng Wang

**Affiliations:** 1 Center for Biosensors and Bioelectronics, The Biodesign Institute, Arizona State University, Tempe, AZ 85287, USA; 2 School of Molecular Sciences, Arizona State University, Tempe, AZ 85287, USA; 3 School of Electrical, Computer and Energy Engineering, Arizona State University, Tempe, AZ 85287, USA; 4 School of Biological and Health Systems Engineering, Arizona State University, Tempe, AZ 85287, USA; 5 Division of Hematology & Oncology, Mayo Clinic Arizona, Phoenix, AZ 85054, USA; 6 Division of Hematology & Oncology, Louisiana State University, New Orleans, LA 70112, USA; 7 John Shufeldt School of Medicine and Medical Engineering, Arizona State University, Tempe, AZ 85287, USA

**Keywords:** chimeric antigen receptor T-cells, CAR T, optical imaging, microfluidic chip, digital counting, whole blood, agglutination, machine learning, object classification

## Abstract

Chimeric antigen receptor (CAR) T-cell therapy is an effective treatment for hematologic malignancies. However, it is limited by high costs, risk of severe toxicities such as cytokine release syndrome and neurotoxicity, and heterogeneous patient responses. The current therapy monitoring depends largely on subjective symptom assessment, routine laboratory tests, and basic vital signs, without real-time, quantitative evaluation of CAR T-cell expansion or activation in clinical practice. This lack of timely immune monitoring hampers individualized care and contributes to increased treatment costs. To address this need, we present a proof-of-concept, label-free rapid optical imaging (ROI) biosensor with automated machine learning analysis for direct quantification of CAR T-cells from whole blood. This microfluidic platform integrates red blood cell (RBC) removal, CAR T-cell capture, and imaging-based quantification on a single chip, eliminating the need for centrifugation, staining, and operator-dependent interpretation. For validation, 50 μL whole blood samples spiked with Jurkat cells expressing CD19 CARs underwent RBC depletion by agglutination and microfiltration. The remaining blood components were then incubated on a sensor chip functionalized with recombinant CD19 protein. Captured CAR T-cells were imaged by brightfield microscopy and automatically enumerated using a machine learning algorithm trained on fluorescence-validated cells. The CD-19 cells’ capture performance was validated by flow cytometry and fluorescence imaging. The trained machine learning model validated at 88% sensitivity and 96% specificity. Buffer and whole blood calibration curves were established across clinically relevant concentrations (1–1000 cells/µL) with triple replicates. The results showed high correlation (0.975 and 0.990 R^2^) between the spiked concentration and the detected CAR T-cells, with a 95% certainty limit of detection (LOD) and quantification (LOQ) of 0.6 and 1.1 cells/µL for spiked buffer, and 14 and 67 cells/µL for spiked whole-blood, respectively.

## 1. Introduction

Chimeric antigen receptor (CAR) T-cell therapy has exhibited impressive outcomes in patients with non-Hodgkin lymphoma and acute lymphocytic leukemia in recent years, receiving FDA approval in 2017 [1,2,3,4]. However, the clinical benefit of CAR T-cell therapy is significantly offset by severe adverse effects whose onset and intensity are closely correlated with CAR T-cell expansion kinetics and systemic cytokine levels. Overall, up to 86% of treated patients develop cytokine release syndrome (CRS), and approximately 67% experience immune effector cell-associated neurotoxicity syndrome (ICANS) [5]. Collectively, these toxicities, compounded by secondary infectious complications arising from treatment-induced immunosuppression, contribute to a non-relapse mortality (NRM) rate of approximately 9–15% [2,4,5,6,7].

Before the injection of CAR T-cells, patients typically undergo lymphodepletion chemotherapy, which lowers the absolute lymphocyte count (ALC) from 570 to 20 cells/µL under the most common regimen, with ALC recovering to near-normal levels (800–900 cells/µL) after 90 days [8]. Clinical monitoring studies have shown that circulating CAR-T-cell levels after infusion vary widely among patients, ranging from low single-digit counts to several hundred cells/µL [9]. Clinical outcomes have been correlated to a 7-day threshold of roughly 19 cells/µL [10]. Based on these data, a full clinical range of 1–1000 cells/µL is desired for research purposes, with the clinical outcome range being 10–500 cells/µL. Since CAR T-cell expression levels vary widely from day-to-day and patient-to-patient, real-time access to objective, quantitative measures of CAR T-cell expansion and cytokine dynamics could markedly enhance patient management by enabling early detection of CRS and neurotoxicity, supporting pre-emptive interventions prior to progression to severe toxicity, and providing prognostic markers of treatment failure to guide timely initiation of additional diagnostics or salvage therapies.

The current assays for monitoring CAR T-cell expansion and cytokine production include flow cytometry and immunoassays. While suitable for research and clinical trial contexts, these approaches are impractical for routine clinical monitoring given their reliance on costly instruments, specialized expertise, batched testing, and processing times of hours to days [2,6,7,11]. As a result, patient care continues to rely on indirect measures such as subjective symptoms, routine laboratory results, and vital signs, particularly fever, while direct quantification of CAR T-cell activity remains limited to correlative trial studies.

This reliance on delayed and nonspecific markers creates a critical “blind spot” in patient monitoring, leading to missed opportunities for early detection of CRS, neurotoxicity, or treatment failure, and limiting precision in clinical decision-making. The paradox is evident: the most advanced and expensive immunotherapy available today is routinely monitored using subjective and indirect clinical parameters that may provide late or misleading information. Beyond clinical challenges, these monitoring gaps contribute significantly to the overall cost of CAR T-cell treatment care, currently estimated at $800,000 to more than $1 million per patient in the United States, driven largely by extended inpatient monitoring and hospitalization [12].

To address these challenges and improve safety, efficacy, and affordability, there is an urgent need for rapid, cost-effective, point-of-care (POC) assays capable of real-time quantification of CAR T-cell expansion and CRS or neurotoxicity-associated cytokine biomarkers in peripheral blood. Access to such objective metrics would enable earlier intervention, support outpatient management, optimize care delivery, and reduce costs while improving patient outcomes.

Recent advances in microfluidic and lab-on-chip technologies highlight the potential of miniaturized blood analysis platforms [13,14,15]; however, their complexity, reliance on specialized materials, and high production costs have limited widespread clinical adoption [16]. Thus, a practical, affordable POC method for monitoring CAR T-cell activity in whole blood remains an unmet need.

Here, we describe the initial development of a simple, POC-compatible diagnostic platform for monitoring CAR T-cell expansion. The approach integrates rapid red blood cell (RBC) depletion with label-free brightfield optical imaging and automated machine learning-based cell identification to directly quantify CAR T-cell concentrations from small volumes of whole blood samples. Validation against fluorescence imaging and flow cytometry confirmed the specificity and accuracy of CAR T-cell detection. This proof-of-concept study focuses on the flow-based assay and data processing, and establishes the feasibility of a low-cost, scalable technology for real-time monitoring of CAR T-cell therapy. The successful development and deployment of this technology will enable earlier detection of treatment failure or toxicities, guide timely interventions, decrease dependence on prolonged hospitalizations, and facilitate safer, more economical outpatient care.

## 2. Materials and Methods

### 2.1. Overview of the CAR T-Cell Detection Workflow

**Figure 1:**
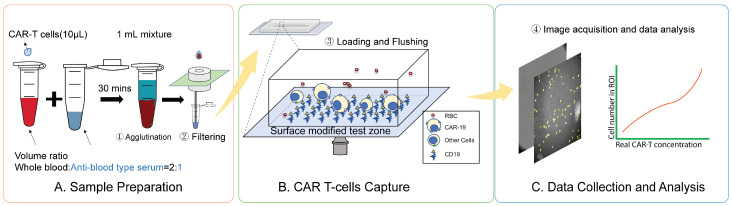
Workflow of the centrifuge-free rapid optical imaging-based CAR T-cell detection method: (**A**) Sample preparation—(1) A small whole blood sample of 50 µL is collected and then spiked with a predetermined concentration of Jurkat CD19 CAR T-cells for calibration curve generation and testing purposes. (2) The whole blood sample is mixed with 25 µL anti-blood-type antibody and left at room temperature to agglutinate for 30 min. (3) The agglutinated sample is then passed through a filter and the fluid is collected through a capillary tube to remove agglutinated RBCs. (**B**) CAR T-cells Capture—(4) The filtered sample is loaded into the functionalized sensor chip and incubated for 15 min to capture CAR T-cells to the sensor surface. (5) Unbound cells are washed away with buffer. (**C**) Data collection and analysis—(6) Optical microscopy images of the captured cells on the sensor chip surfaces are collected. (7) These images are processed to count Jurkat CD19 CAR T-cells. (8) Jurkat CD19 CAR T-cell concentration in the blood samples is determined using a calibration curve.

Figure 1 summarizes the streamlined workflow for CAR T-cell detection. The protocol consists of four critical stages: RBC depletion, CAR T-cell capture, imaging, and data analysis. First, we remove RBCs from whole blood samples without centrifugation or RBC disruption procedures. Instead, the whole blood is mixed with RBC agglutination antibody solution for 30 min to allow the RBCs to aggregate, and a membrane filter with a pore size larger than white blood cells filters out the aggregated RBCs. Next, the filtered blood is loaded onto a microfluidic sensor chip functionalized with a recombinant CD19 protein to facilitate the specific capture of CD19 CAR T-cells via receptor interaction. After 15 min of incubation, nonspecifically attached cells are removed through washing cycles. The remaining cells are recorded through digital capture of brightfield microscopy images and processed for automated cell identification and counting.

### 2.2. Sample Delivery and Detection System

As depicted in Figure S1, the sample delivery and detection system consists of four parts: **(1)** Flow control—Syringe pump (Chemyx Fusion 100, Chemyx Inc., Stafford, TX, USA, Catalog #: 07100) provides constant flow rate (10 µL/min); 6-port injection valve (VICI Valco Instruments Co. Inc., Houston, TX, USA, #08N-0232H) for switching between washing buffer and the fluorescent protein; 20 mL Sterile syringe for loading washing buffer; and 1 mL sterile syringe for loading cells samples (VWR # 53548-008 and 53548-001, VWR International, Radnor, PA, USA). **(2)** Sensor chip—Homemade microfluidic chips with a straight channel and a CD19-functionalized surface for CAR T-cell capture and imaging. **(3)** Image acquisition—An inverted microscope (Olympus IX81, Olympus Corporation of the Americas, Center Valley, PA, USA, 40×/0.75 NA objective) with a CMOS camera for recording brightfield and fluorescence image sequences. **(4)** Data processing—Digital cellular crowding was reduced through a custom Python 3.9 script; CAR T-cells were identified and quantified with ilastik, a machine learning-based image processing toolkit.

### 2.3. Microfluidic Chip Fabrication

The microfluidic chip included 4 components (Figure S2). From the bottom to the top, there was a 25 × 50 mm coverslip (Mercedes Medical Glass Coverslips, Fisher Scientific, Waltham, MA, USA, #15-184-03), a microfluidic channel defined by two layers of double-sided tapes (4 Mil/101.6 μm thick for each layer; #3M 444, 3M, St. Paul, MN, USA), top cover glass (Cole-Parmer, Vernon Hills, IL, USA, 22 × 40 mm, #UX-48511-10) with two 1 mm diameter pre-drilled holes as the inlet and outlet, and two polydimethylsiloxane (PDMS, Momentive Specialty Chemicals, Waterford, NY, USA; #RTV-615-044) channel connector holders attached to the top cover slides via oxygen plasma treatment. The microfluidic channel defined by the double-sided tape was designed in Adobe Illustrator (2020, Adobe Inc., San Jose, CA, USA) and fabricated with a laser cutter (Universal Laser System, VLS 4.15, Universal Laser Systems Inc., Scottsdale, AZ, USA). The channel was linear, with 203 μm in height, 1.0 mm in width, 35 mm in length, and a total volume of 7.1 μL. The PDMS channel connector holders were fabricated with a classic protocol [17,18,19]. 30 mL mixed polydimethylsiloxane was poured into a clean plastic Petri dish with a flat surface, degassed in a vacuum desiccator (for 30 min), and baked in an oven at 60 °C for 60 min. Next, the hardened PDMS was peeled off from the dish and cut into small cubes. A hole was punched through each PDMS cube with a hole puncher (Robbins Instruments Inc., Chatham, NJ, USA, # RBP-075) to hold the stainless-steel flow channel connectors. The chip was assembled bottom up, starting with a functionalized bottom glass, followed by attaching the double-sided tape-defined channel. Then the top cover slide with drilled holes was aligned with the channel end and attached to the tape. Finally, two PDMS holders were aligned with the top glass holes and attached.

### 2.4. Surface Chemistry

The surface modification process of the sensor chip is depicted in Figure S3. CD19 recombinant protein was immobilized onto the sensing surface (the top surface of the bottom glass) using (3-glycidyloxypropyl) trimethoxylsilane (GPTMS) (Sigma-Aldrich St. Louis, MO, USA; #440167) as a coupling agent [20,21,22,23]. The oxirane ring in the coupling agent undergoes a nucleophilic addition reaction with the amino group in the target protein, leading to covalent binding of the protein to the glass surface [22,24]. The coverslips (22 × 50 mm) were cleaned with 99% isopropanol (VWR #: 67-63-0, VWR International, Radnor, PA, USA) and deionized water (15 MΩ/cm, 0.22 μm filtration) and air-dried using a nitrogen stream. The coverslips were further cleaned using oxygen plasma (Harrick Plasma, Ithaca, NY, USA, PDC-001 # PDC001051102 High frequency, 2.96 W, 3 min). The 1% (*v*/*v*) GPTMS in anhydrous isopropanol (IPA) was freshly prepared one day before starting the surface modification. The plasma-treated glass coverslips were soaked in the GPTMS solution overnight (24 h) at room temperature in a glass staining jar, then cleaned with anhydrous IPA and distilled water, and dried under a nitrogen stream. The top coverslip and PDMS connector holders were also cleaned using the same washing steps. After the sensor chip was assembled, 0.1 µg/µL human recombinant CD19 protein solution (Sino Biological US Inc., Houston, TX, USA; #11880-H08H), diluted in pre-dissolved 50 mM sodium carbonate-bicarbonate buffer (pH = 9.6) (Medicago, 09-8922-100), was injected into the channel via the inlet and incubated for 30 min. The chip was gently washed with 1 mL 1× live cell imaging solution (Thermo Fisher Scientific, Waltham, MA, USA, #A14291DJ) and incubated with 1× blocking reagent (10%, *v*/*v*, Roche Diagnostics Corporation, Indianapolis, IN, USA; #11096176001) statically for 30 min at room temperature to block nonspecific binding sites. The as-prepared chip was used for CAR T-cell measurement within 6 h.

### 2.5. Sample Preparation

To determine the total blood cell count, fresh whole blood samples were diluted 1000-fold with live-cell imaging solution to ensure the cell counter’s maximum reading threshold was not exceeded. To prepare the Jurkat T-cell samples, 2 mL each of wild-type (WT) Jurkat T-cells and Jurkat CD19 CAR T-cells were centrifuged at 300 *g* for 5 min. The supernatant was discarded, and the cells were re-diluted in live cell imaging solution to eliminate potential interference from FBS and other culture medium components. The concentration of all the cell samples was adjusted to ~2 × 10^3^ cells/µL as a standard cell sample before any experiment. This standard sample was then spiked into live cell imaging solution or human whole blood to final concentrations of 10^3^, 10^2^, 10^1^, and 10^0^ Jurkat CD19 CAR T-cells/µL. Two types of blood samples were used: reagent RBCs purchased from Immucor Inc., Norcross, GA, USA (Catalog #: IG2338), and fresh human whole blood collected from healthy volunteers using K_2_ EDTA tubes (BD, Franklin Lakes, NJ, USA, #23-021-015) (approved by IRB STUDY00008255). The whole-blood spiked samples were designed to mimic clinical patient samples.

**Figure 2:**
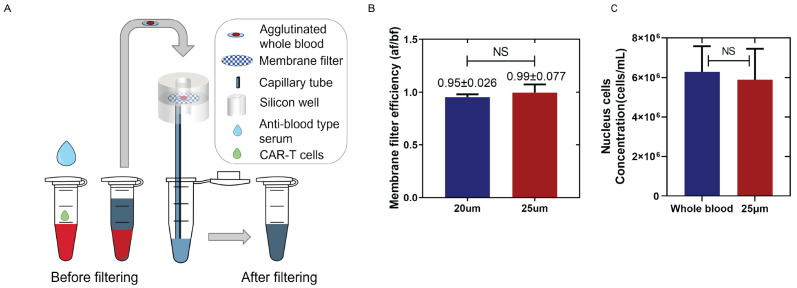
Principle and result of centrifuge-free RBCs removal from whole blood: (**A**) Schematic of agglutinated sample filtration for removing RBCs from the whole blood sample. (**B**) Comparison of the membrane filter efficiency between 20 µm and 25 µm membrane filters. The filtering efficiency is the ratio of WBC concentration after and before passing through the membrane filter (Efficiency = WBC_after_/WBC_before_). Data was generated using LUNA II cell counter (**C**) comparing the WBCs’ concentration in the undiluted whole blood samples (before filtering) and the agglutinated and filtered (25 µm filter) samples. The hemacytometer assay was applied. The standard deviation of triplicate tests determines the error bars (mean ± SD).

To remove RBCs in the blood samples, 25 µL of anti-blood-type solution (Ortho Clinical Diagnostic Inc., Raritan, NJ, USA; # 6901934) was gently mixed with 50 µL of whole blood or reagent RBCs spiked samples at room temperature for 30 min. The agglutinated samples were then transferred to a simple filter system to remove any aggregated RBCs (Figure 2A). A hydrophilic membrane filter (Sterlitech Corporation, Kent, WA, USA, #PCT25013100) with a 25 µm pore size was inserted between two reusable silicone sample wells (FlexiPERM micro12, Sarstedt, Nümbrecht, Germany, #94.6011.436). A capillary tube (VitroCom Inc., Mountain Lakes, NJ, USA, #8270) located below the filter provided the capillary force to extract the liquid portion of the agglutinated sample containing non-agglutinated cells for measurement. A total of 50 ± 5 µL of filtered samples can be collected after the agglutination and filtering process.

### 2.6. Sample Loading and Processing

The filtered sample was introduced into the sensor chip through a standard PDMS inlet connection, which is assembled with a 21-gauge blunt needle (Flow-Tips, Jensen Global Inc., Santa Barbara, CA, USA, G.21/0.8 mm, PBN-GRN-200) plus a 1 mL syringe (BD, 309628, Franklin Lakes, NJ, USA). The CD19 protein immobilized on the chip surface selectively captured the target Jurkat CD19 CAR T-cells. After 15 min of incubation, a washing buffer (live cell imaging solution; 20 mL syringe (BD, 302830, Franklin Lakes, NJ, USA)) was perfused through the microfluidic system at a flow rate of 10 µL/min for 1 min to remove non-target cells from the region of interest. Two sets of images were acquired for each experiment: one before and one after the washing step. In total, each set consisted of 30 images covering the entire length of the linear channel.

### 2.7. Optical Image Acquisition

An inverted microscope (Olympus IX81, 40×/0.75 NA objective) with a CMOS camera (Hamamatsu ORCA-Flash 4.0 V3, Hamamatsu Corporation, Bridgewater, NJ, USA; pixel size: 6.5 μm; view size: 2048 × 2048 pixels) was used to obtain brightfield and fluorescent images. A mercury light (Olympus U-LH100HG, Olympus Corporation of the Americas, Center Valley, PA, USA) and a filter set (excitation wavelength: 543 ± 10; emission wavelength: 593 ± 40) were used for fluorescence imaging. The brightfield images were recorded with a 0.1 s exposure time, while the fluorescence images were recorded with a 4 s exposure time. Optical images were captured in 16-bit monochrome format.

### 2.8. Cell Culture

This study used two cell lines, Jurkat CD19 CAR T-cells and corresponding WT Jurkat T-cells. Jurkat CD19 CAR T-cells were engineered to express the anti-human CD19 protein, containing a single-chain variable fragment (scFv) derived from the FMC63 monoclonal antibody, which mimics the function of human CAR-19+ T-cells. On the other hand, the WT cells do not express the CAR-CD19 molecule. Both cell lines were cultured and prepared in accordance with the previously published protocol [25]. Specifically, the cells were cultured in Nunc EasYFlask 75 cm^2^ (Thermo Fisher Scientific, 156472, Waltham, MA, USA) using RPMI 1640 medium (Gibco, Thermo Fisher Scientific, Grand Island, NY, USA, A1049101), supplemented with 10% (*v*/*v*) fetal bovine serum (FBS) (Gibco, A31606-02) and 1% (*v*/*v*) penicillin–streptomycin (Corning Incorporated, Corning, NY, USA, 30-002-CI). Cell density was maintained at 1 × 10^3^ cells/µL.

### 2.9. Cell Counting with a Cell Counter

An automated cell counter (Logos Biosystems Inc., Annandale, VA, USA, LUNA-II^TM^, L40001) was used as a reference method to count cell density in the samples. For proper counting using the cell counter, the Jurkat CD19 CAR T-Cells were rendered permeable with 0.1% Triton X-100, Sigma-Aldrich, St. Louis, MO, USA (9036-19-5), and nuclei were visualized using 0.4% trypan blue, Thermo Fisher Scientific, Waltham, MA, USA (15250061) as a DNA indicator [26,27].

### 2.10. Immunostaining

Immunostaining was performed to verify the identity of the Jurkat CD19 CAR T-cells. The immunostaining protocol was adapted from the manufacturer’s instructions and standard cell immunostaining protocols [28]. Briefly, 1 mL of the cells (concentration: 1 × 10^3^ cells/µL) in a culture medium was transferred into a 15 mL centrifuge tube and centrifuged at 300 *g* for 5 min. The supernatant was discarded, replaced with the same amount of living cell imaging solution, and re-centrifuged. The cells were resuspended in 1 mL 1× blocking reagent (10% *v*/*v*) for 30 min of blocking. The cells were centrifuged again, and the supernatant was aspirated. The cells were then incubated with Human CD19-phycoerythrin (PE)-conjugated protein (1:50 dilution; Acro Biosystems, Newark, DE, USA, #CD9-HP2H3) for 30 min at room temperature in the dark. The centrifugation process was repeated twice with 1 mL of living cell imaging solution to wash off unbound CD19-PE. Finally, 50 μL of the stained cell samples were pipetted onto a clean microscope slide attached with a reusable silicone sample well (FlexiPERM micro12 #94.6011.436, Sarstedt, Nümbrecht, Germany) and covered with a microscope coverslip. The sample was observed under a fluorescent microscope with the focal plane adjusted to the center of the cells to minimize background (Figure S5).

To validate the identity of the Jurkat CD19 CAR T-cells captured in a microfluidic chip, PE-conjugated human CD19 protein was injected into the chip system after flushing the unbound cells, incubated for 30 min, and washed for 1 min at 10 µL/min to remove unbound protein prior to imaging.

### 2.11. Flow Cytometry

The flow cytometry process was conducted according to a standard protocol [29,30]. Briefly, 1 × 10^6^ cells were centrifuged at 300 *g* for 5 min, washed 3 times with live cell imaging solution, and counted using the Logos LUNA-II cell counter (Logos Biosystems, Anyang-si, Republic of Korea) to verify cell concentration. The buffer-diluted Jurkat WT/CD19 CAR T-cells were then blocked with 1000 μL 0.5% BSA (Sigma Aldrich # 05470, St. Louis, MO, USA) for 30 min, followed by triplicate centrifuging and washing steps. Subsequently, the cells were incubated with 100 μL PE-conjugated human CD19 protein (1:50) for 30 min. The cells were centrifuged and washed three times to remove excess fluorescent protein, then analyzed on an Attune NxT Flow Cytometer (Thermo Fisher Scientific, Waltham, MA, USA).

### 2.12. Image Processing and Analysis

**Figure 3:**
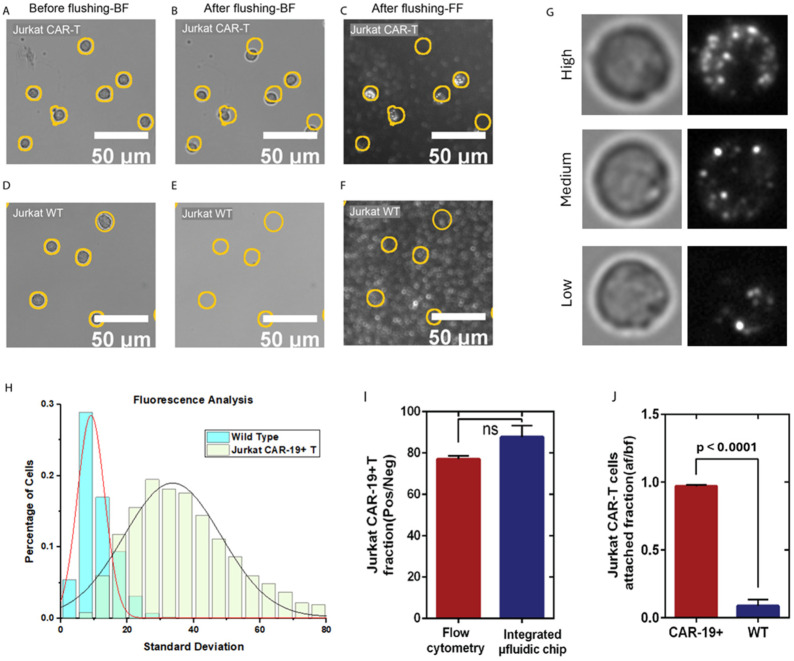
Validation of CD-19-captured Jurkat CD19 CAR T-cells with fluorescent staining and flow cytometry. Panels (**A**–**F**) are representative brightfield images of the Jurkat CD19 CAR T-cells (**A**,**B**) and Jurkat wild-type T-cells (**D**,**E**) before (**A**,**D**) and after (**B**,**E**) flushing. Panels (**C**,**F**) are fluorescence images of the CD19-PE-stained cells after the flushing step. Yellow circles mark the locations of attached cells before flushing. Panel (**G**) is brightfield (Left) and corresponding fluorescence images (right) of three CAR T-cells with low, medium, and high CD-19 expression levels, where clustered fluorescence signals are clearly visible. Graph (**H**) is the histogram showing the statistical distribution of the fluorescence spectrum. Fluorescence spot statistics are represented by the standard deviation of the fluorescence image divided by the median of the image to normalize across different experiments and slight background brightness variations. The histogram contains analysis of over 3000 fluorescently stained Jurkat CD19 CAR T-cells and Jurkat wild-type T-cells. Panel (**I**) is a comparison of the percentage of identified CAR T-cells in the cultured Jurkat CD19 CAR T-cells line by flow cytometry (77.0 ± 1.7%) and fluorescent images of the attached cells (87.7 ± 5.7%), with error bars representing the standard deviation of three or more replicates. No significant difference was observed between the two methods. Panel (**J**) is the fraction of the Jurkat CD19 CAR T-cells attached to the surface, calculated as the ratio of the number of cells attached after and before flushing. Error bars represent the standard deviation of three or more replicates, and a significant difference was observed between the two groups (*p* < 0.0001).

Each experimental trial consisted of multiple independent imaging regions of interest in the capture zone along the PDMS channel. Training and validation images were recorded in each trial before and after flushing, and after fluorescent staining to compare the captured cell positivity ratio. The recorded images in the experimental datasets (Figure 3A–F, Figures S5, S7 and S8) were analyzed using Fiji ImageJ 1.51w [31], and the fluorescence and whole-blood trials (Figure 3G,H and Figures S9–S11) were analyzed with Python 3.9 and ilastik (EMBL, Heidelberg, Germany) 1.4.0 [32,33]. Fluorescence distribution statistics were gathered by recording the locations of the CAR T-cells in brightfield imaging and using region-of-interest data to analyze corresponding fluorescent images to extract both fluorescence intensity and spatial information.

**Figure 4:**
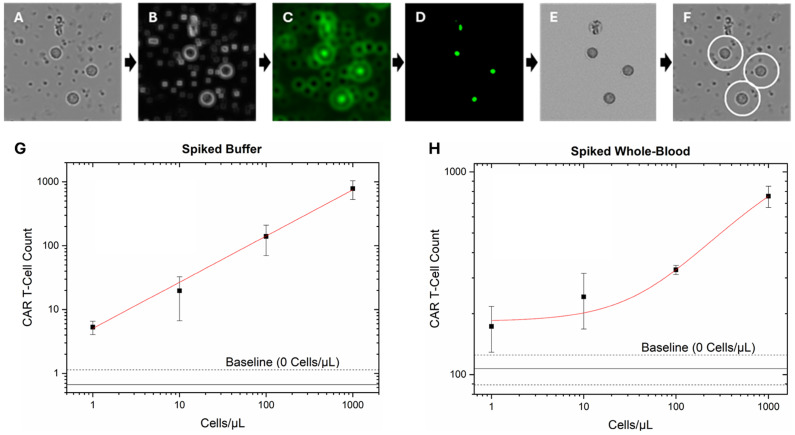
Automated algorithm overview and results of algorithm output using spiked whole blood at varying CAR T-cell concentrations: (**A**) Section of a brightfield image representing ~0.1% of the total captured image area. (**B**) Localized standard deviation filter. (**C**) Modified match filtering. (**D**) Negative value thresholding. (**E**) Identification of local maxima and radial segmentation of the original image. (**F**) Identification of CAR T-cells (circled in white) using the ilastik ML-based software. (**G**) Output of automated algorithm analyzing buffer spiked with 4 different concentrations of CAR T-cells with power fitting, R^2^ value of 0.975. Fitting details are shown in Figure S11. (**H**) Output of automated algorithm analyzing whole blood spiked with 4 different concentrations of CAR T-cells with logarithmic fitting with an R^2^ value of 0.990. Fitting details are shown in Figure S12.

Whole-blood CAR T-cell spiked trials, used for calibration curve, were analyzed using an automated Python pre-filtering algorithm paired with a machine learning (ML) based (bio)image segmentation and classification software, ilastik. (Figure 4). The prefiltering algorithm consists of: (1) images subjected to a localized standard deviation filter; (2) a modified match filter; (3) negative value thresholding; (4) local maxima identification; and (5) radial segmentation of the original brightfield image to identify and separate potential CAR T-cell candidates for categorization using ilastik. This unique prefilter eliminates issues caused by cellular crowding and enhances segmentation algorithms by removing most platelets and separating each potential target from its neighbors (Figure S8). ilastik uses an interactive GUI (Graphic User Interface) to manually label objects into different categories, allowing the program to analyze the chosen pixels for various statistics, for use in a 100-tree Random Forest classifier. To train this model, 4 commonly occurring objects in the brightfield image datasets were chosen as categories: CAR T-cells verified by fluorescent tagging; platelets; RBCs; and cellular debris/anomalous/broken cells (categorized as “Other”) (Figure S10). The training and testing sets consisted of 150 samples per category in a 100/50 training/test split, with the results given in Figure S14. CAR T-cell category utilized fluorescence imaging verified cells (10 images, 15 cells per image), while the other categories utilized whole-blood samples chosen by visual appearance (6 images, 25 per image). All the training images were outside the experimental group used for whole-blood spiked calibration and subjected to the Python prefiltering algorithm beforehand.

## 3. Results

### 3.1. Centrifuge-Free, Agglutination-Based RBCs Removal

The ratio of RBCs to white blood cells (WBCs) in whole blood is approximately 600:1–8, making RBC removal a critical step for effective capture and quantification of CAR T-cells [34]. Classic centrifugation-based RBC removal methods [35,36,37,38] are not POC compatible. Ammonium Chloride Potassium (ACK) lysing buffer can lyse RBCs [39,40,41], but the efficiency is low (~60%) and affects white blood cell viability [42]. Microfluidic RBC removal methods have been explored, including filtration [43,44,45,46,47,48], dielectrophoresis [49,50,51,52,53], or geometrical channels [54,55,56,57]. However, these designs often increase the complexity and cost of the assay [16].

To accomplish RBC removal in a cost-effective, POC-compatible manner, we implemented a simple agglutination and filtration method for centrifuge-free RBC removal from the whole blood samples. By inducing antibody-mediated RBC agglutination followed by filtration through a large-pore membrane, the aggregated RBCs were eliminated while preserving the WBCs and other nucleated cells. For proof-of-concept testing, the whole blood was incubated with the corresponding anti-blood-type serum for 30 min and then passed through a simple filtration system containing a polycarbonate track-etched (PCTE) filter (Figure 2A). Additional details are mentioned in Section 2.5. Agglutinated RBCs were retained on the filter, and the remaining blood components were collected via capillary force into a test tube. Given the average size of lymphocytes (7 to 15 µm) [58], PCTE filters with pore sizes of 20 µm and 25 µm were evaluated for recovery efficiency using buffer-diluted Jurkat CD19 CAR T-cells and Jurkat wild-type cells. Concentrations of T-cells were measured pre- and post-filtration (Figure 2B). Both membranes achieved >95% recovery and were statistically similar. The larger-pore-size (25 µm) filter was chosen for the remainder of the study to reduce the potential loss of CAR T-cells.

The efficiency of the filtration method in the whole blood samples spiked with the Jurkat CD19 CAR T-cells was further assessed using hemocytometer counting as the reference standard (Figure S4). The WBC counts before and after filtration showed no significant difference compared with undiluted whole blood (*p* = 0.6353), confirming that this POC-compatible method effectively removes RBCs while maintaining a high WBC recovery rate (Figure 2C).

### 3.2. CAR T-Cell Capture, Detection, and Validation

The surface chemistry used to functionalize the sensor surface for Jurkat CD19 CAR T-cell capture is shown in Figure S3, with details provided in Section 2. The samples were introduced to the CD19-functionalized sensor surface and incubated for 15 min to allow Jurkat CD19 CAR T-cell capture, followed by washing with live cell imaging solution to remove nonspecifically bound cells.

Heterogeneous CAR expression in transfected Jurkat CD19 CAR T-cells is a known issue that can affect the precision and accuracy of CAR T-cell detection, as illustrated in Figure 3G, which shows an expected Gaussian distribution of expression levels [59,60]. This results in a range of receptor levels naturally leading to differing binding strengths to the CD19-coated surface. A deeper understanding of expression levels and cell functionality is needed to improve treatment effectiveness [60,61,62].

To evaluate capture efficiency and specificity, the Jurkat CD19 CAR T-cells (positive for CAR expression) and Jurkat WT T-cells (negative control) were tested (Figure 3). The flow cytometry confirmed the CD19 CAR expression levels, and capture efficiency on the CD19-coated surface was validated by fluorescence staining of the captured cells [61,62]. Following the washing step, 97% of the Jurkat CD19 CAR T-cells remained bound to the sensor surface (Figure 3A,B,J), with positive fluorescent signals observed in 88% of the captured cells (Figure 3C,G–I). This fraction was marginally higher than the 77% CD19 CAR population measured by the flow cytometry (Figure 3I and Figure S6) because of the preferential retention of the CD19 CAR T-cells on the CD19-functionalized surface. In contrast, only 8.9% of the Jurkat WT T-cells remained attached after washing (Figure 3D,E,J), indicating a low level of nonspecifically bound cells post-washing (Figure 3F,I and Figure S6).

Statistical analysis of ~1500 cells from each line, tested independently in duplicate assays, was performed by paired evaluation of brightfield and fluorescence images. Although brightfield morphology appeared similar across the cells, the fluorescence imaging revealed CAR expression on the Jurkat CD19 CAR T-cell surface, with clustered CD19 signals apparent as bright dots (Figure 3G). The quantitative analysis of fluorescence intensity, normalized by dividing pixel intensity standard deviation by the median for each cell, revealed two distinct Gaussian distributions separating the Jurkat CD19 CAR T-cells and WT T-cells (Figure 3H; details in Table S1). Notably, approximately 12% of the Jurkat CD19 CAR-T-cells exhibited low CAR expression (categorized using the intersection point of Figure 3H), phenotypically resembling the higher fluorescence WT T-cells and possibly not contributing to the effective CD19 CAR T-cell population. Insight into CD-19 expression levels and cellular functionality would be an important addition to the current CAR T-cell profiling for treatment effectiveness [60,61].

Overall, these results demonstrate that the CD19-functionalized surface effectively captures Jurkat CD19 CAR T-cells, as evidenced by a higher percentage of fluorescently positive cells among the captured Jurkat CD19 CAR T-cells compared to the flow cytometry counts (88% vs. 77%). These findings confirm the robustness and specificity of the CD19 capture surface for the accurate detection of Jurkat CD19 CAR T-cells without relying on fluorescence markers.

### 3.3. CAR T-Cell Quantification in Whole Blood

As the ultimate goal is to build towards a point-of-care (POC) diagnosis, experiments were conducted using CAR T-cell-spiked whole blood. The whole blood samples produced substantial nonspecific adherence of non-CAR T-cells to the sensor surface, primarily platelets, unfiltered RBCs, and other leukocytes or debris. While the washing step effectively removed CAR-19-negative T-cells and RBCs (Figure 3A–F and Figure S7), it was less effective against platelets and other blood components. This led to cell crowding and contamination, impairing data processing and hampering cell segmentation. This resulted mainly in false negatives that required manual parameter adjustments, creating implicit bias and failing to meet POC automation requirements.

To address this limitation, we developed a label-free, bright-field imaging-based data processing algorithm for automated identification and quantification of Jurkat CD19 CAR T-cells. The algorithm, implemented in Python and integrated with the machine learning platform ilastik, operates in two stages: (1) broad identification of cells within a relevant size range and morphology (Figure 4A–E) and (2) automated classification and counting of the identified cells to yield CAR T-cell quantification (Figure 4F). ilastik was chosen for its relatively simple, well-known ML method based on calculable statistics and its ease of use, providing a solid foundation for future development.

System performance was evaluated by spiking Jurkat CD19 CAR T-cells at four concentrations into undiluted human whole blood from healthy donors. After washing, captured cells across the full channel (30 regions of interest) were digitally captured and enumerated by the algorithm, with non-spiked whole blood as a baseline/control. Each concentration was tested in triplicate to assess reproducibility, and representative images are shown in Figure S8. A standard calibration curve was generated by plotting the number of algorithm-identified Jurkat CD19 CAR T-cells against their known input concentrations in both buffer (Figure 4G) and spiked whole blood (Figure 4H). The performance of this ML method was evaluated using 400 training and 200 test samples, yielding 88% sensitivity and 96% specificity for CAR T-cell identification. Full performance results and metrics are shown in Figure S14. The calibration curves yielded 95% confidence limits of detection (LOD) and quantification (LOQ) of 0.6 and 1.1 cells/µL for spiked buffer trials (Figure S11) and 14 and 67 cells/µL for whole-blood trials (Figure S12). The buffer trials show that the ML method provides high-correlation power, with fitting that covers the comprehensive research clinical range of quantification. The whole-blood trials required a logarithmic fitting with a higher detection limit due to a high background (>100 cell count). This background is caused by the white blood cell in the healthy-donor blood, which will likely not be an issue for real patient samples, as discussed in detail below.

## 4. Discussion

This proof-of-concept study establishes the feasibility of a simple microfluidic chip for quantifying CAR-T-cells. It is the first step towards building a low-cost, scalable, POC platform for real-time monitoring during CAR T-cell therapy. The technology has the potential to lower therapy costs by addressing critical gaps in the current patient management. Clinical outcomes and toxicities are closely linked to CAR T-cell expansion and the dynamics of immune activation-associated cytokines, both of which govern therapeutic response and the development of serious, and sometimes life-threatening, complications such as CRS and neurotoxicity. Despite their clinical relevance, these parameters are not routinely measured due to the high cost, procedural complexity, and slow turnaround times inherent to conventional assays such as flow cytometry and multiplex cytokine immunoassays (Table S2) [2,6,7,11].

POC devices quantifying CAR T-cell and cytokine profiles in real time could give clinicians objective information not available under the current routine vital sign and intermittent laboratory testing regimens. Recent advances [63] in microfluidic and lab-on-chip technologies have demonstrated the feasibility of CAR T-cell functional assessment [64], cytokine analysis [65], and low-sample-volume assays [66], and their adoption in both research and early translational settings continues to expand. As most existing platforms remain optimized for discrete, laboratory-based measurements rather than longitudinal clinical monitoring, their integration into routine care pathways remains limited. There remains a need for point-of-care approaches that can support timely clinical decision-making, particularly for early, precise detection of functional cell-level or impending treatment failure. Given that prolonged hospitalization and monitoring can account for a significant portion of the >$800,000 per-patient cost of CAR T-cell therapy, reducing reliance on inpatient care could yield substantial cost savings across the healthcare system.

The described platform, which integrates centrifuge-free RBC removal, label-free optical imaging, and automated machine learning-based CAR T-cell detection, represents a significant advance towards practical POC patient monitoring. The system demonstrated the capability to capture and identify CAR T-cells at clinically relevant concentrations in whole blood within 1 h, at a material cost of approximately $5 per test (Table S3). This platform has demonstrated a full clinical research measurement range for spiked buffer samples.

However, spiked healthy-donor blood shows a higher limit of detection level due to the higher background in blank, which is likely caused by capture of CD-19+ B-cells that are naturally present in blood and indistinguishable from T-cells by our current image processing algorithm. This high background issue should be mitigated when using patient samples, as lymphodepletion lowers blood lymphocytes 50–250-fold [8], which would expectably lower the background caused by CD-19+ B-cells by the same scale. Alternatively, when sufficient clinical data are available, the ML method could be trained to distinguish between the B-cells and CAR-T-cells. To ensure the consistent performance of the ML method in clinical samples, we need to train and validate the algorithm using clinical samples and use independent evaluation data separate from training data. These tasks will be a primary goal for future clinical studies.

While type A blood from a donor was used in this proof-of-concept study, the agglutination-based RBC removal strategy is not inherently restricted to type A samples. In clinical practice, a patient’s ABO blood type is routinely determined before therapeutic procedures, including CAR-T-cell therapy. Therefore, antibody selection can be readily tailored to the patient’s blood group, which should work in 93% of the population, except for type O-negative. Agglutination antibodies are widely available and affordable compared to more specific antibodies. Alternatively, an RBC-specific membrane protein, such as Band-3, can be used as a universal agglutination target, which is the most abundant RBC membrane protein [67].

This work provides foundational evidence supporting the future clinical translation of rapid, deployable biosensors for CAR T-cell monitoring. Beyond the scope of this work, ongoing development towards a POC device will implement a miniature fluid control system [68] and a portable imaging system, and extend the platform to multi-analyte cytokine profiling. Future work to develop better training data with known truths and additional cell categories (Monocytes and lymphocytes) will allow training and comparison of multiple cutting-edge ML methods. Further testing in a clinical setting using finger-prick capillary samples in real-world patient cohorts will be compared against the current medical standards. Collectively, these innovations may redefine monitoring strategies for cellular immunotherapies, improve clinical safety, drive down the cost of care, and expand access to these life-saving treatments.

## 5. Patents

The technology described in this article is protected by a US patent application (18/778,278) filed by the Arizona Board of Regents on behalf of Arizona State University.

## Data Availability

The prefilter Python code, trained ilastik ML model, and example dataset images are available at https://github.com/rmporte2-asu/CAR-T-cell-Data-Processing.git (accessed on 27 February 2026). Raw data, including the full training and validation dataset for this study, are available at https://doi.org/10.5281/zenodo.18868456.

## Supplementary Materials

The following supporting information can be downloaded at https://www.mdpi.com/article/10.3390/bios16050240/s1. Figure S1: Illustration of the flow-driven detection setup; Figure S2: Schematic figure of the fluidic chip structure; Figure S3: Sensor surface modification protocol; Figure S4: Standard cell counting images with a hemacytometer; Figure S5: Image processing workflow utilizing Fiji software; Figure S6. Flow cytometry matching results; Figure S7: Post-flush images of Jurkat CD19 CAR T-cells and wild-type Jurkat cells in varying conditions; Figure S8: Representative images of five concentrations used for the calibration experiment; Figure S9: Illustration of cellular crowding effects from whole-blood trials on segmentation and prefiltering performance enhancements; Figure S10: Machine learning categories representative images; Figure S11: Spiked buffer trials details, fittings, and statistics; Figure S12: Spiked whole-blood trials details, fittings, and statistics; Figure S13: Python Prefilter Parameters; Figure S14: Machine Learning performance metrics; Table S1: Statistical fitting details; Table S2: Comparison of the current representative direct CAR-T-cell counting approach; Table S3: Unit price for one chip summary. Reference [69] is cited in Supplementary Materials.

## Abbreviations

The following abbreviations are used in this manuscript:

ACK	Ammonium Chloride Potassium
CAR	Chimeric Antigen Receptor
CRS	Cytokine Release Syndrome
FBS	Fetal Bovine Serum
FDA	Food and Drug Administration
GPTMS	(3-glycidyloxypropyl) trimethoxylsilane
IPA	Isopropanol
ML	Machine Learning
PCTE	Polycarbonate Track-Etched
PDMS,	Polydimethylsiloxane
PE	Phycoerythrin
POC	Point-Of-Care
ROI	Rapid Optical Imaging
RBC	Red Blood Cell
WBC	White Blood Cells
WT	Wild Type
