# Supplementary material for: Machine Learning-Assisted Rapid Optical Imaging for Label-Free CAR T-Cell Detection in Whole Blood
Source: Biosensors (Basel). 2026 Apr 24;16(5):240. doi: 10.3390/bios16050240 (PMC13204493; doi:10.3390/bios16050240)
Supplement: Supplementary file 1 [file biosensors-16-00240-s001.zip › biosensors-4134718-supplementary.pdf]

Supporting Information

Table of contents

Figure S1. Illustration of the flow-driven detection setup
Figure S2. Schematic figure of the fluidic chip structure
Figure S3. Sensor surface modification protocol
Figure S4. Standard cell counting images with a hemacytometer
Figure S5. Image processing workflow utilizing Fiji software
Figure S6. Flow cytometry matching results
Figure S7. Post-flush images of Jurkat CD19 CAR T-cells and wild type Jurkat cells
Figure S8. Representative images of five concentrations used for the calibration experiment
Figure S9. Illustration of cellular crowding effects from whole-blood trials on segmentation and prefiltering performance enhancements
Figure S10. Machine learning categories representative images
Figure S11. Spiked buffer trials data and statistics
Figure S12. Whole-blood trials details, fittings, and statistics
Figure S13. Python Prefilter Parameters
Figure S14. Machine learning performance metrics
Table S1. Statistical fitting details for Figure 3H
Table S2. Comparison of the current representative direct CAR-T cell counting approach
Table S3. Unit price for one chip summary

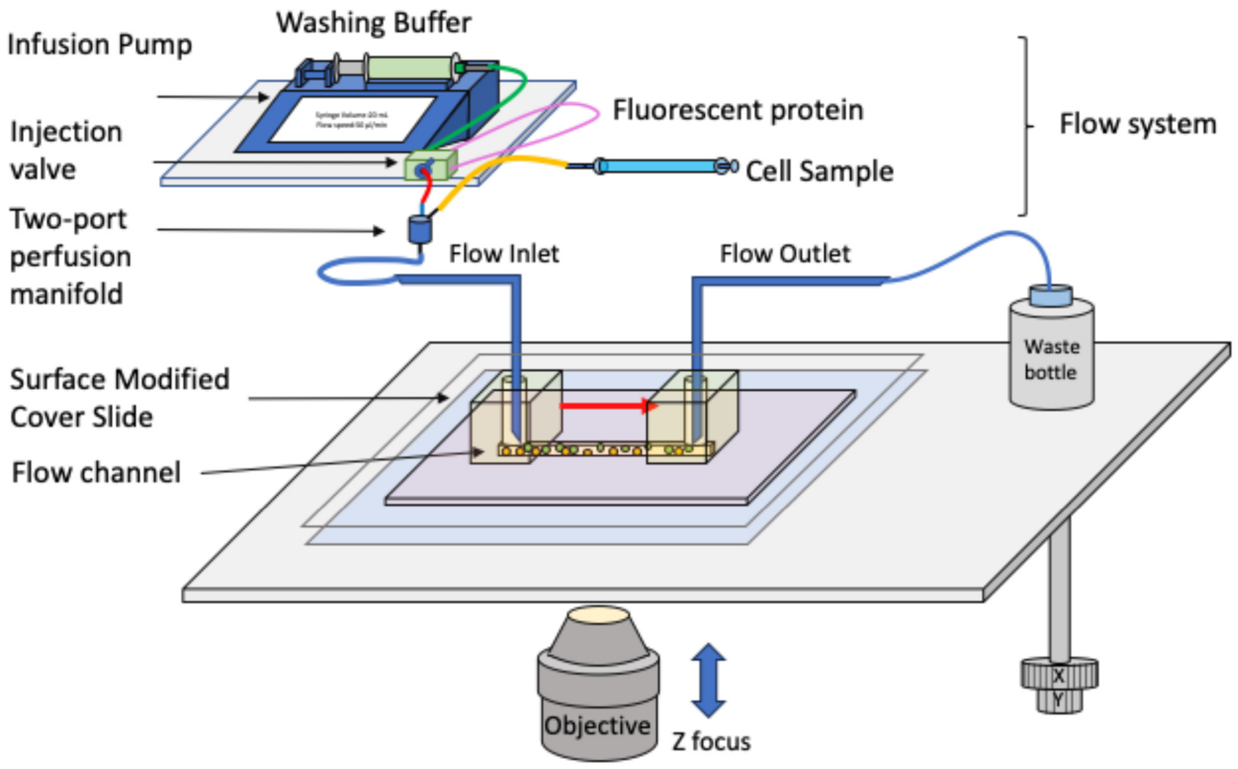

Figure S1. Schematic of the CAR-T cell detection setup composed of three sections: a flow system, a microfluidic chip, and an imaging system. The flow system includes a motorized syringe pump, an injection valve, a manifold, and tubing for controlled injection of samples and buffer. The homemade fluidic chip with a simple straight channel is used for CAR T-cell capture for imaging. Bright-field and fluorescence images are recorded using an Olympus IX-81 inverted microscope equipped with a Hamamatsu CMOS camera.

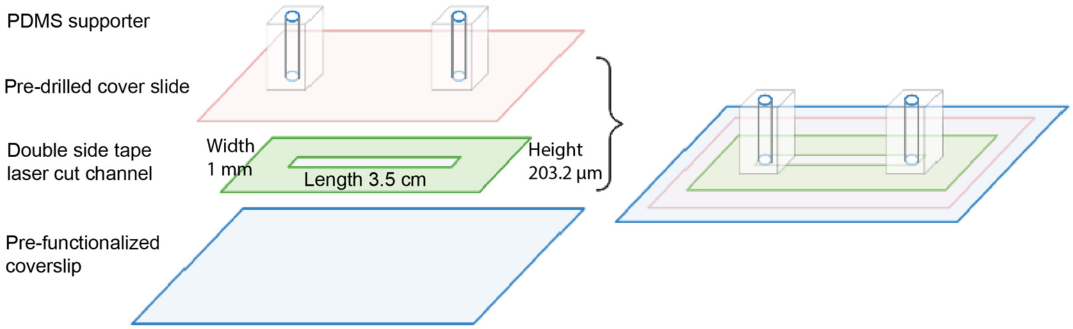

Figure S2. Schematic figure of the fluidic chip structure. Our homemade fluidic chip contains three layers and four components, which include one 25 x 50 mm coverslip, one laser-cut channel made from two layers of double-sided tape, a pre-drilled cover slide with two holes used as the inlet and

outlet, and two connection needle supporters made with polydimethylsiloxane (PDMS) for supporting the injection tubes' needles. Channel dimensions are 35 x 1 x 0.2 mm.

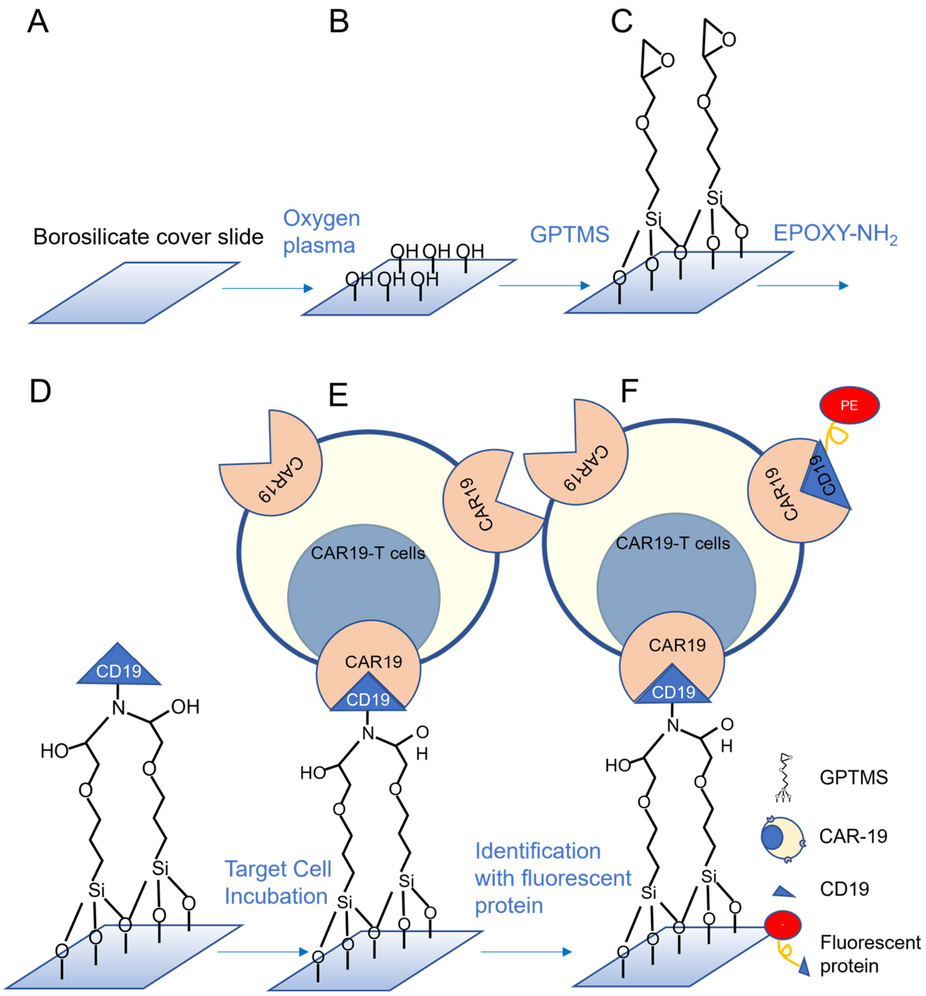

Figure S3. Sensor surface modification protocol. A) Clean the coverslip with oxygen plasma to introduce hydroxyl groups on the surface. B) Submerge the coverslip into 1% (v/v) GPTMS solution to perform epoxy-functionalization via the silanization reaction[1, 2]. C) Incubate and immobilize the CD19 protein on the pre-modified coverslip through covalent Epoxy-amino conjugation[3] , forming the capturing protein structure. D) Immobilize the target cell through the CD19: CAR-19 binding procedure. E) Apply CD19 PE-conjugated protein for fluorescent staining verification.

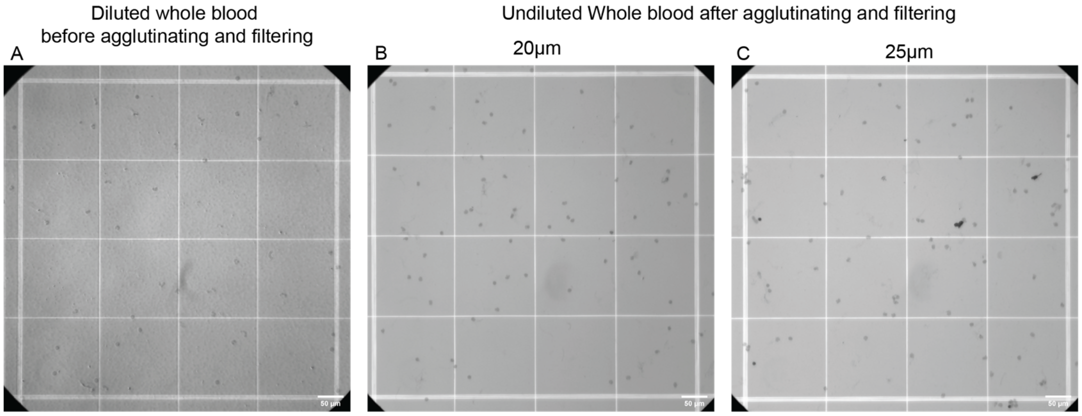

Figure S4. Standard cell counting results with a hemacytometer of different samples. Panel A) is diluted whole blood before agglutinating and filtering (Dilution factor: 8). Panel B) and C) are undiluted whole blood after agglutinating and filtering with 20 μm or 25 μm pore sizes filter processing (Dilution factor: 6).

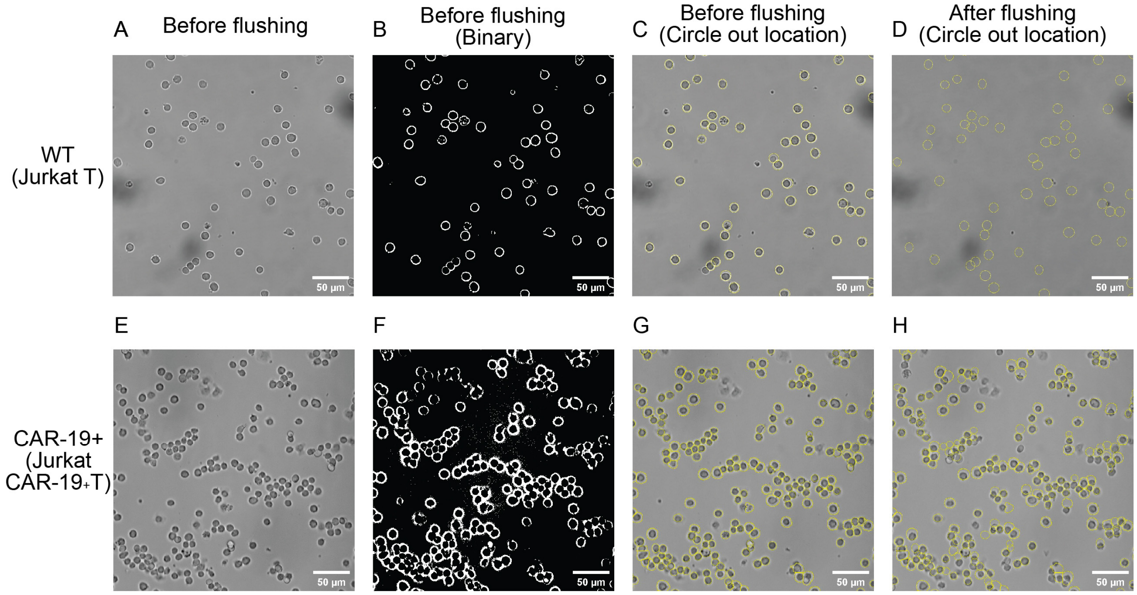

Figure S5. Initial image processing workflow utilizing Fiji software to evaluate the fraction of cells that remain attached following a flushing step, brightfield images used for processing were captured in 16-bit monochrome format. Panels A and E depict brightfield images of cell samples prior to flushing, whereas Panels B and F demonstrate binary images obtained through Fiji processing. By utilizing the "Particle counting" and "Add to manager" functions in Fiji, the location of each cell can be recorded and saved. Subsequently, the location file can be overlaid onto the post-flushing images (Panels D and H) to identify cells within the region of interest. This approach leverages the original cell locations to assess their attachment fraction after the flushing step.

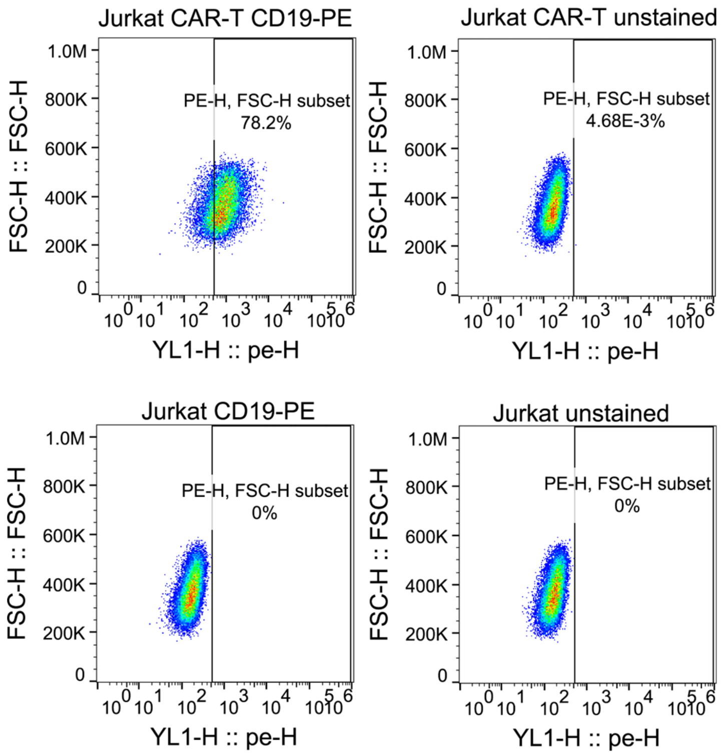

Figure S6. Flow cytometry matching results indicate that 77% of the Jurkat CAR-T group cells were considered Jurkat CD19 CAR T cells (top left plot), while the wild-type Jurkat T cells or unstained cells show only background fluorescence.

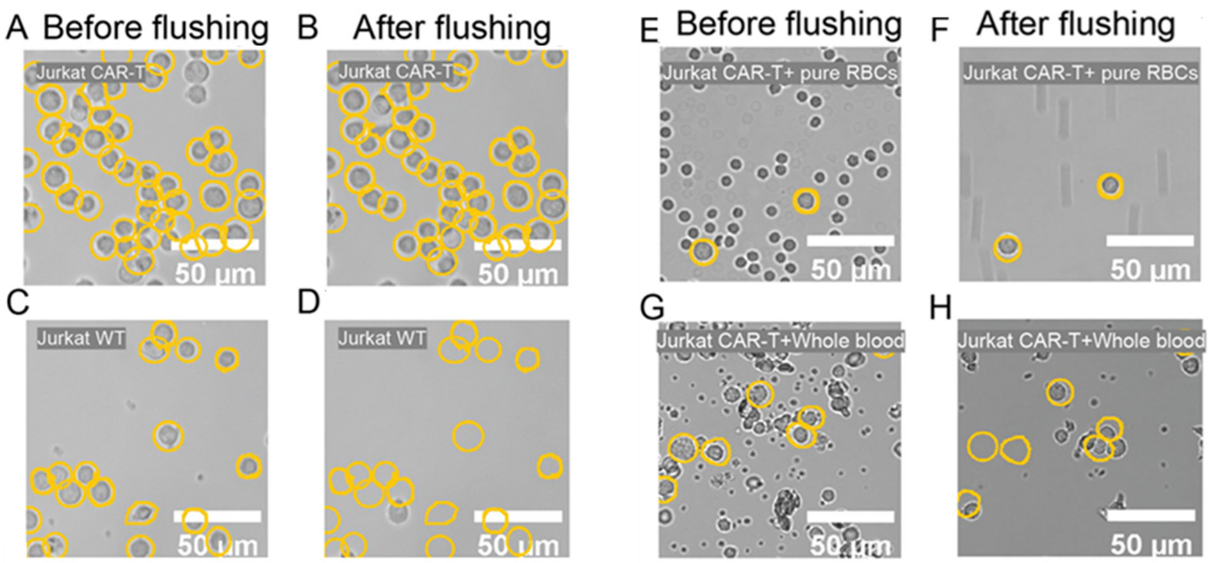

Figure S7. Post-flush images of Jurat CD19 CAR and wild-type T-cells in varying conditions captured by the recombinant CD19-coated surface. Panels show representative brightfield images

before and after the flushing step of (A, B) pure Jurkat CD19 CAR T-cells, (C, D) pure Jurkat wild-type cells, (E, F) Jurkat CD19 CAR T-cells spiked in RBCs, and (G, H) whole blood.

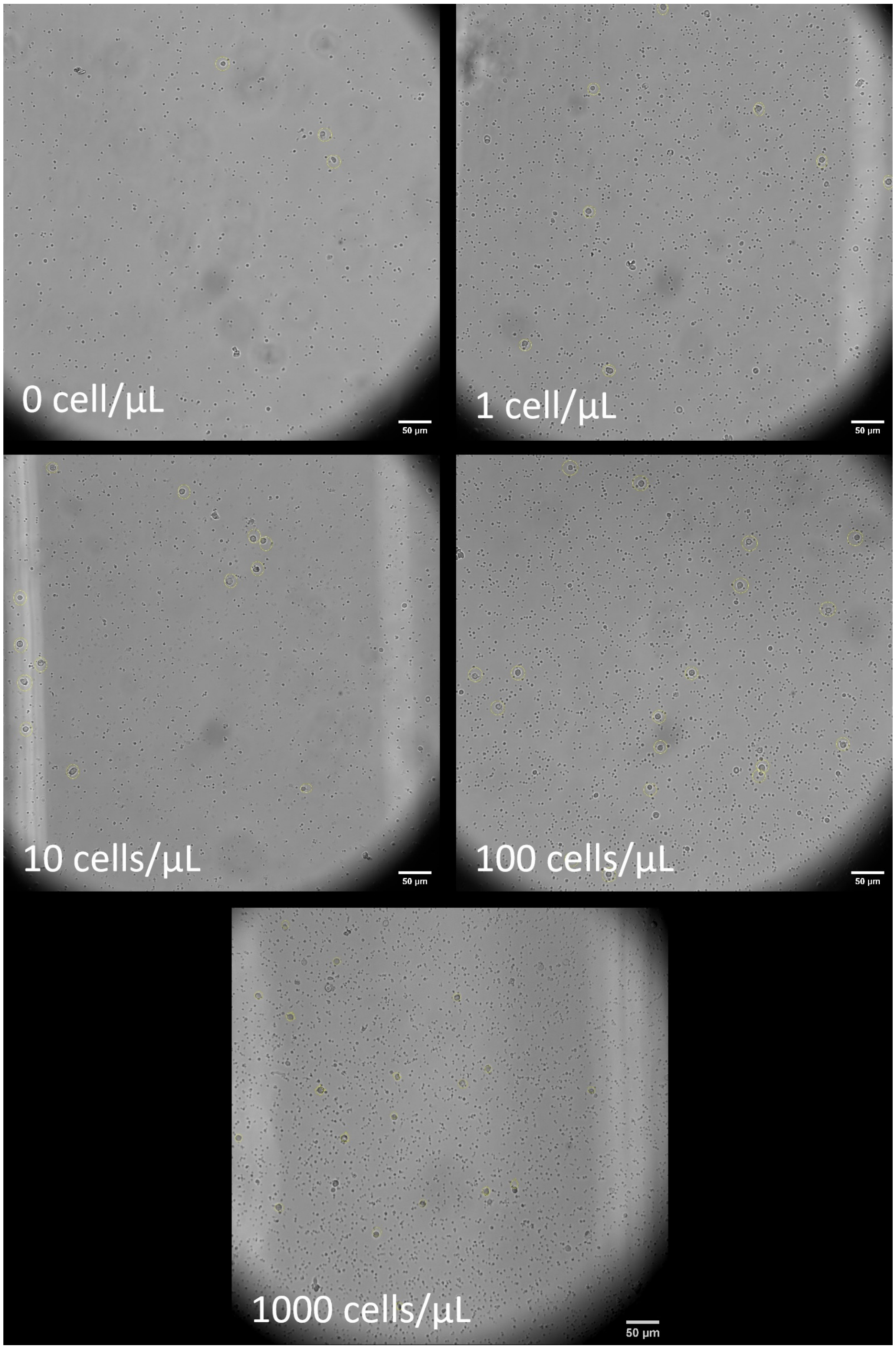

Figure S8. Representative images of five Jurkat CD19 CAR T-cell concentrations used for the calibration experiment. Representative brightfield images of one of the 30 images recorded in each

of the three duplicate experiments at the five CAR T-cell concentrations after flushing (Scale bar: 50μm).

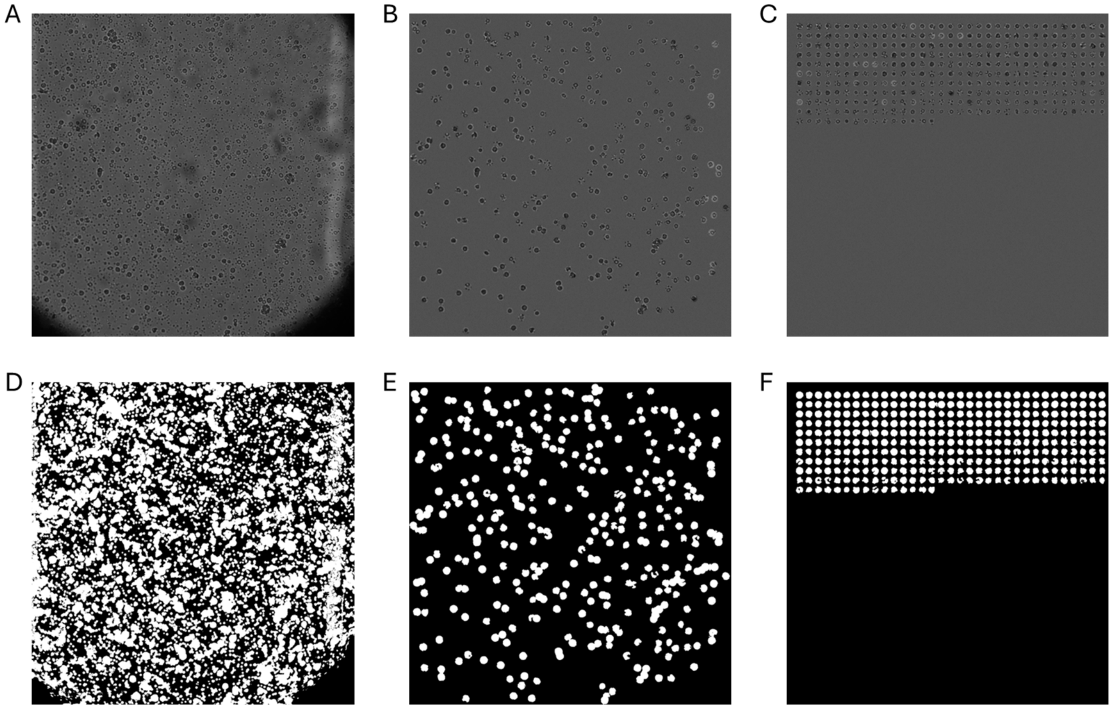

Figure S9. Cellular crowding effects in the whole blood brightfield image (A-C), and the effect of the prefiltering algorithm on cell segmentation (D-F). Black and white in D-F indicates the backgrounds and objects predicted by ilastik. (A) raw image and (D) segmentation result, most objects are touching others and are therefore counted as a single object for classification. (B) filtered out non-CAR T-cell candidates, (E) touching candidates, ranging from 2-10 cell candidates, are counted as a single object for classification. (C) and (F) candidates are moved and evenly spaced to eliminate errors due to touching cells.

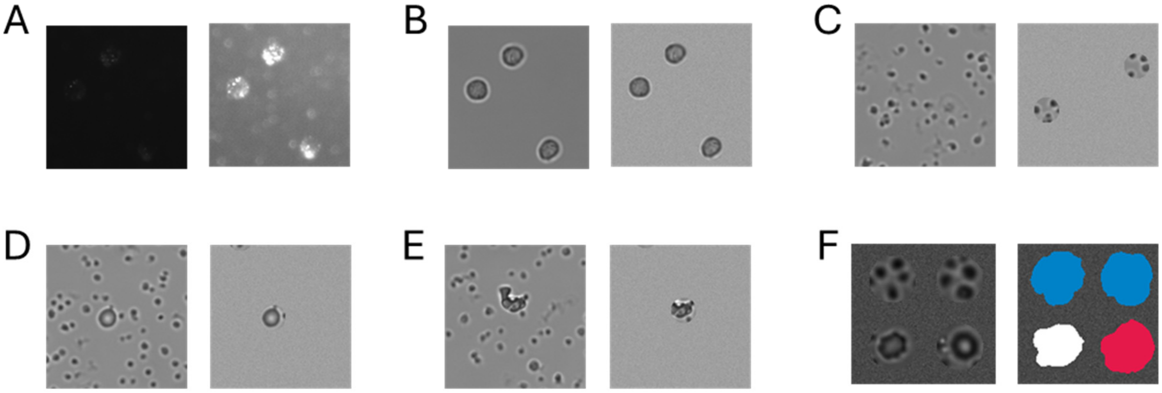

Figure S10. Training dataset examples. Each individual cell is considered a sample. (A) Examples of fluorescence image (cropped raw image and contrast-enhanced) of tagged CAR T-cells. (B-E)

Examples of cropped raw brightfield images and post-filtered images used for model training. (B) Category 1: CAR T-cells in buffer verified by fluorescence. (C) Category 2: Platelets (normally multiple touching cells grouped due to segmentation difficulties and most individual platelets removed by the prefilter). (D) Category 3: Red blood cells. (E) Category 4: Other (cellular debris and/or anomalous cells). (F) Example of manually labeling segmented images into categories using ilastik. The training data strategy is labeling the most visually obvious cells: blue (platelets), red (RBC), and white (unlabeled).

A

Concentration (cells/μL)	Mean CAR T-cell Count	Standard Deviation	Coefficient of Variation (%)
**0**	0.7	0.5	70
**1**	5.3	1.2	23
**10**	19.7	13.0	66
**100**	139.7	69.8	50
**1000**	784.7	254.3	32
		**Average CV**	48

B

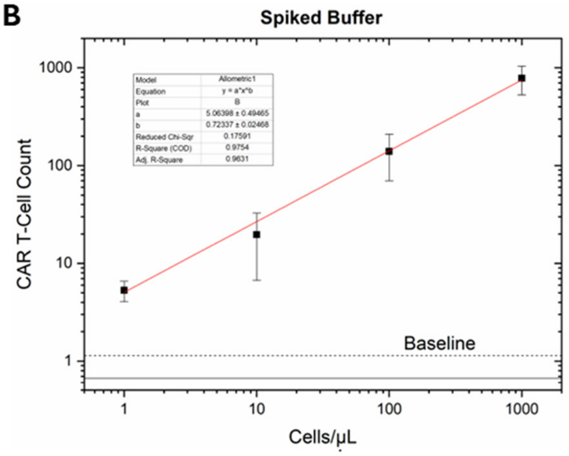

C

Statistic	Formula	Results (Count)	Result (Cells/μL)
**Limit of Blank**	$LoB = mean_{blank} + 1.645(SD_{blank})$	1.4	N/A
**Limit of Detection**	$LoD = LoB + 1.645(SD_{1\ cell/\mu L})$	3.5	0.6*
**Limit of Quantification**	$LoQ = mean_{blank} + 10(SD_{blank})$	5.4	1.1*

Figure S11. Spiked buffer trials data and statistics. (A) Mean, standard deviation, and coefficient of variation for each spiked concentration with 3 replicates. (B) Power fitting of all non-baseline concentrations. (C) Calculated Limit of Blank, Detection, and Quantification based on experimental results. *Units converted based on model fitting.

A

Spiked Concentration (cells/µL)	Mean CAR T-cell Count	Standard Deviation	Coefficient of Variation (%)
**0**	107	18	17
**1**	173	44	26
**10**	242	74	30
**100**	329	17	5
**1000**	759	91	12
		**Average CV**	18

B

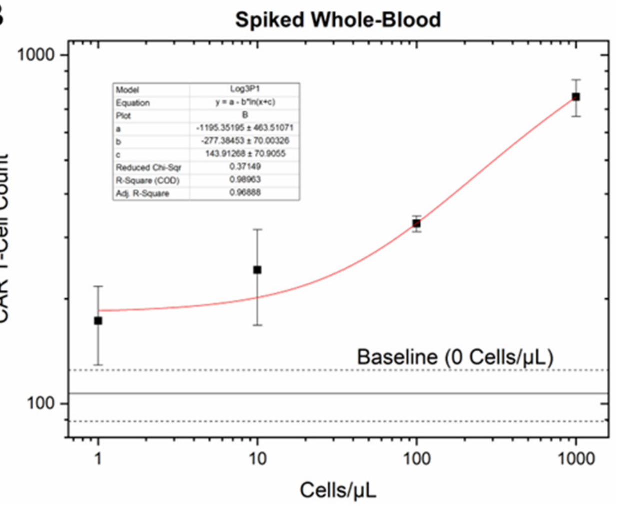

C

Statistic	Formula	Results (Count)	Result (Cells/µL)
**Limit of Blank**	$LoB = mean_{blank} + 1.645(SD_{blank})$	137	N/A
**Limit of Detection**	$LoD = LoB + 1.645(SD_{1\ cell/\mu L})$	209	14*
**Limit of Quantification**	$LoQ = mean_{blank} + 10(SD_{blank})$	287	67*

Figure S12. Spiked whole-blood trials data and statistics. (A) Mean, standard deviation, and coefficient of variation for each spiked concentration with 3 replicates. (B) Logarithmic fitting of all non-baseline concentrations. (C) Calculated Limit of Blank, Detection, and Quantification based on experimental results. *Units converted based on model fitting.

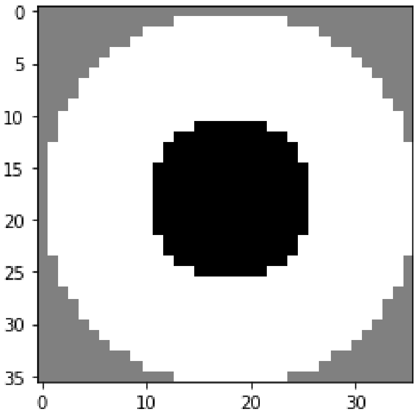

Figure S13. Python Prefilter Parameters. Captured 12-bit images converted to 8-bit for ilastik computational purposes. 2248x2248 images padded to 2448x2448. Local Standard Deviation Kernel: 5. Matching Kernel. Values: Black = -1; white = 1; gray = 0. Local Maxima Minimum Distance: 20. Local Maxima Threshold: 0.6. Full code and example images provided at: https://github.com/rmporte2-asu/CAR-T-cell-Data-Processing.git.

A

Predicted Category	*CAR T-Cells*	*Platelets*	*Red Blood Cells*	*Other*
1	44	0	5	3
2	0	41	13	2
3	4	9	30	2
4	2	0	2	43

B

	CAR T-Cell	*Platelets*	*Red Blood Cell*	*Other*
True Positive	44	41	30	43
False Positive	6	9	20	7
True Negative	192	185	185	193
False Negative	8	15	15	7
Sensitivity (%)	88.0	82.0	60.0	86.0
Specificity (%)	96.0	92.5	92.5	96.5

Figure S14. Python performance metrics for the trained ilastik Machine Learning model. (A) Cell truths and predicted categories, based on 100/50 split training/testing set per category. (B) Performance statistics per trained category.

Table S1. Statistical fitting details for Figure 3H (A,B). Analysis was completed using OriginPro.

A

Model	Gauss
Equation	y=y0 + (A/(w*sqrt(pi/2)))*exp(-2*((x-xc)/w)^2)
Plot	D
y0	9.21666E-4 ± 0.0012
xc	9.06717 ± 0.16622
w	8.20232 ± 0.34951
A	2.91749 ± 0.10687
Reduced Chi-Sqr	9.68513E-5
R-Square (COD)	0.94458
Adj. R-Square	0.94213

B

Model	Gauss
Equation	y=y0 + (A/(w*sqrt(pi/2)))*exp(-2*((x-xc)/w)^2)
Plot	E
y0	0.00233 ± 0.00116
xc	33.62675 ± 0.42194
w	29.21092 ± 0.88147
A	6.85755 ± 0.19367
Reduced Chi-Sqr	7.58047E-5
R-Square (COD)	0.96792
Adj. R-Square	0.96651

Table S2. Comparison of the current representative direct CAR-T cell counting approach

**Example Method**	**BD FACSCanto II Flow Cytometer**	**VWR® Automated cell counter Fluo**	**POC ROI platform**
**Platform**	Fluorescence + Algorithm	Fluorescence + Algorithm	Optical Imaging* + Algorithm
**Portability (weight, kg)**	196 kg	~8kg	<3 kg (Anticipated)
**Sample source**	Blood draw required	Blood draw/ Finger-pricked blood	Finger-pricked blood
**Centrifuge-necessity**	√	√	
**Detection Sample**	Plasma/serum	1000x diluted whole blood/pure cell lines	50 µL finger prick whole blood
**Labor-necessity**	Well-trained technician	Middle-trained automatic readout	Simple-trained automatic readout
**Sample to Result**	At least 3 hours	5~15 mins	30 mins max
**Low Detection Limit**	0.1-100 cells/mL (restricted by sample volume)	10 cells/µL	~1 cell/µL
**Platform Cost ($)**	$23,995.00 (used)	$4,325.62	Anticipated cost $1,000*

*Optical imaging platform spec and cost are based on a POC image acquisition and analysis platform that is currently under development and testing.

Table S3. Unit price for one chip summary

**Materials**	**Source**	**Unit Price for one chip ($)**
Substrate cover slide	Fisher Scientific #15-184-03	0.06 (1 slide)
Top cover slide	Cole-Parmer #UX-48511-10	0.21 (1 slide)
Double side tape	3M # 444	0.75 (3 cm × 1 cm)
PDMS	Momentive Specialty Chemicals #RTV-615-044	0.0045 (0.0.1% (v/v))
GPTMS	Sigma-Aldrich #440167	0.0051 (0.0075%(v/v))
Isopropanal	VWR #67-63-0	1.64 (3.75%(v/v))
Capture antibody	Sino Biological #11880-H08H	2.34 (1 μg)
Washing buffer	Thermo Fisher Scientific #A14291DJ	0.058 (1 mL)
Blocking reagent	Roche #11096176001	0.079 (0.1% (v/v))
**Total price ($)**		5.15

References:

1. Ojea-Jiménez, I.; Urbán, P.; Barahona, F.; Pedroni, M.; Capomaccio, R.; Ceccone, G.; Kinsner-Ovaskainen, A.; Rossi, F.; Gilliland, D., Highly Flexible Platform for Tuning Surface Properties of Silica Nanoparticles and Monitoring Their Biological Interaction. *ACS Applied Materials & Interfaces* **2016,** *8* (7), 4838-4850.
2. Ghanbari, A.; Attar, M. M., A study on the anticorrosion performance of epoxy nanocomposite coatings containing epoxy-silane treated nano-silica on mild steel substrate. *Journal of Industrial and Engineering Chemistry* **2015,** *23*, 145-153.
3. Mateo, C.; Torres, R.; Fernández-Lorente, G.; Ortiz, C.; Fuentes, M.; Hidalgo, A.; López-Gallego, F.; Abian, O.; Palomo, J. M.; Betancor, L.; Pessela, B. C. C.; Guisan, J. M.; Fernández-Lafuente, R., Epoxy-Amino Groups: A New Tool for Improved Immobilization of Proteins by the Epoxy Method. *Biomacromolecules* **2003,** *4* (3), 772-777.
